# The Efficacy of the Quorum Sensing Inhibitor FS8 and Tigecycline in Preventing Prosthesis Biofilm in an Animal Model of Staphylococcal Infection

**DOI:** 10.3390/ijms140816321

**Published:** 2013-08-07

**Authors:** Oriana Simonetti, Oscar Cirioni, Federico Mocchegiani, Ivana Cacciatore, Carmela Silvestri, Leonardo Baldassarre, Fiorenza Orlando, Pamela Castelli, Mauro Provinciali, Marco Vivarelli, Erika Fornasari, Andrea Giacometti, Annamaria Offidani

**Affiliations:** 1 Clinic of Dermatology, Department of Clinical and Molecular Sciences, Università Politecnica delle Marche–Ospedali Riuniti, Ancona 60020, Italy; E-Mail: a.offidani@ospedaliriuniti.marche.it; 2 Clinic of Infectious Diseases, Department of Biomedical Sciences and Public Health, Università Politecnica delle Marche–Ospedali Riuniti, Ancona 60020, Italy; E-Mails: o.cirioni@univpm.it (O.C.); carmelasilvestri@libero.it (C.S.); p.castelli@libero.it (P.C.); a.giacometti@univpm.it (A.G.); 3 Centre for Abdominal Surgery and Organ Transplant, Università Politecnica delle Marche–Ospedali Riuniti, Ancona 60020, Italy; E-Mails: f.moccheggiani@univpm.it (F.M.); m.vivarelli@univpm.it (M.V.); 4 Department of Pharmacy, Università degli Studi G. D’Annunzio, Chieti-Pescara 66013, Italy; E-Mails: cacciatore@unic.it (I.C.); baldassarre@unic.it (L.B.); fornasari@unic.it (E.F.); 5 Experimental Animal Models for Aging Units, Research Department, I.N.R.C.A. I.R.R.C.S., Ancona 60100, Italy; E-Mails: f.orlando@libero.it (F.O.); m.provinciali@libero.it (M.P.)

**Keywords:** lipopeptides, tigecycline, FS8, vascular graft infection, bacterial biofilm

## Abstract

We investigated the efficacy of tigecycline and FS8, alone or combined, in preventing prosthesis biofilm in a rat model of staphylococcal vascular graft infection. Graft infections were established in the back subcutaneous tissue of adult male Wistar rats by implantation of Dacron prostheses followed by topical inoculation with 2 × 10^7^ colony-forming units of *Staphylococcus aureus*, strain Smith diffuse. The study included a control group, a contaminated group that did not receive any antibiotic prophylaxis, and three contaminated groups that received: (i) intraperitoneal tigecycline, (ii) FS8-soaked graft, and (iii) tigecycline plus FS8-soaked graft, respectively. Each group included 15 animals. The infection burden was evaluated by using sonication and quantitative agar culture. Moreover, an *in vitro* binding-study was performed to quantify the how much FS8 was coated to the surface of the prosthesis. Tigecycline, combined with FS8, against the adherent bacteria showed MICs (2.00 mg/L) and MBCs (4.00 mg/L) four-fold lower with respect to tigecycline alone in *in vitro* studies. The rat groups treated with tigecycline showed the lowest bacterial numbers (4.4 × 10^4^ ± 1.2 × 10^4^ CFU/mL). The FS8-treated group showed a good activity and significant differences compared to control group with bacterial numbers of 6.8 × 10^4^ ± 2.0 × 10^4^ CFU/mL. A stronger inhibition of bacterial growth was observed in rats treated with a combined FS8 and tigecycline therapy than in those that were singly treated with bacterial numbers of 10^1^ CFU/mL graft. In conclusion, the ability to affect biofilm formation as well, its property to be an antibiotic enhancer suggests FS8 as alternative or additional agent to use in conjunction with conventional antimicrobial for prevention of staphylococcal biofilm related infection.

## 1. Introduction

Healthcare-associated infections (HAIs) are defined as infections not present at the time the patient’s healthcare begins, but which arise afterwards [1]. Unfortunately, HAIs are an increasing problem in modern healthcare because they are costly to the health service and to patients and despite current attempts to resolve the problem, HAI still causes significant patient mortality [1].

*S. aureus* is also one of the most common pathogens in biofilm related infections of indwelling medical devices [2–4]. Bacteria can attach to the surface of biomaterials or tissues and form a multilayered structure consisting of bacterial cells enclosed in an extracellular polymeric matrix [5]. Bacterial behavior within biofilms is regulated by the phenomenon of quorum sensing (QS) [6,7]. Once formed, biofilms may be up to 1000 times more resistant to antimicrobial agents than planktonic cells alone making them particularly difficult to eliminate [8,9]. Several compounds have been used in many *in vitro* and *in vivo* biofilm models [10–14], but none of them significantly eradicate bacterial colonization. Moreover, the widespread use of antibiotics has led to the emergence of multidrug-resistant bacterial strains [15,16]. Tigecycline, a derivate of minocycline, is a glycylcycline that exhibits potent activity against a broad spectrum of bacteria, including staphylococci. Recent reports have shown synergistic effect when tigecycline was combined with other clinically used antibiotics or antimicrobial peptides [17–19]. A novel strategy to prevent biofilm formation is based on the interference with the bacterial cell-cell communication that leads to the virulence phenotype. It is well known that bacteria communicate their cell density by the production of chemical signals in a process known as quorum sensing [20]. This type of signaling allows the bacteria to coordinate the expression of particular genes necessary for their survival. In the case of staphylococci and e.g., *Pseudomonas aeruginosa*, quorum-sensing systems regulate biofilm architecture and detachment, as well as production of toxins [21–23].

*S. aureus* virulence can be inhibited by the heptapetide RNA-III inhibiting peptide (RIP, corresponding to the sequence H-Tyr-Ser-Pro-Trp-Thr-Asn-Phe-NH_2_) by interfering with the known function of staphylococcus quorum sensing [24,25]. RIP is able to inhibit the *in vivo* formation of staphiloccocal biofilm and the agr-regulated toxins production [26]; moreover it shows also synergic effect with most of the common antibiotics used against *S. aureus* infections [13]. Recently, we started a RIP structure-activity relationship study to identify the contribution of each amino acid to its activity synthesizing a series of single alanine mutants of RIP (data not yet published). Alanine is often selected for such scans because it is assumed that single alanine substitutions do not disrupt the secondary structure of the peptide [27]. Among the seven RIP derivatives synthetized, the quorum-sensing inhibitor FS3 (corresponding to the sequence H-Tyr-Ala-Pro-Trp-Thr-Asn-Phe-NH_2_)—obtained substituting the serine residue with alanine in position 2 from the RIP—resulted to enhance the daptomycin efficacy in a rat model of staphylococcal infection [28].

**Figure 1:**
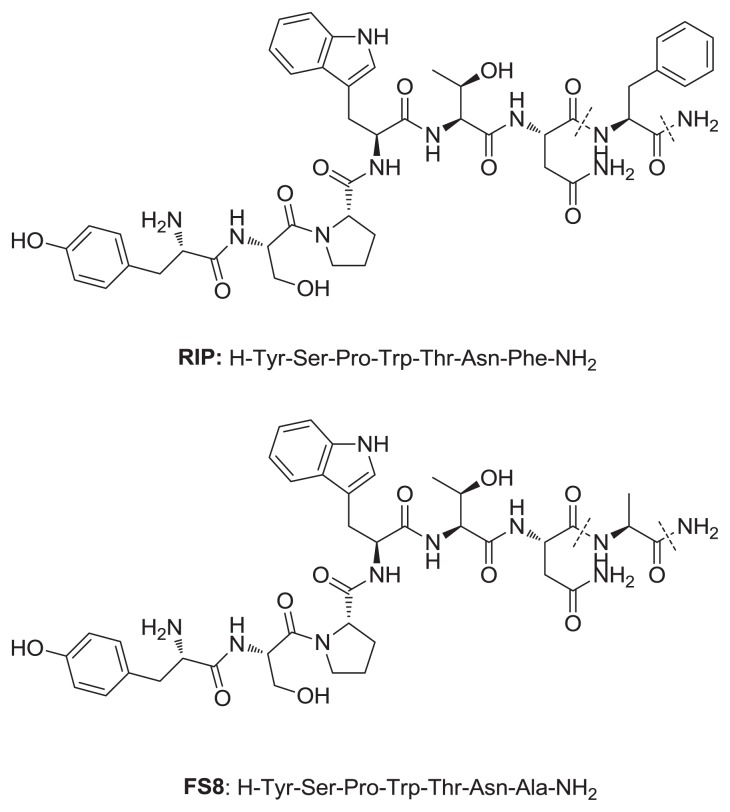
Chemical structures of RIP and FS8.

In the present study, we have investigated the efficacy of tigecycline and FS8 (a RIP derivative, corresponding to the sequence H-Tyr-Ser-Pro-Trp-Thr-Asn-Ala-NH_2_, obtained by substituting the phenylalanine residue with alanine in position 7 from the RIP, (Figure 1) alone or combined in preventing prosthesis biofilm in a rat model of staphylococcal vascular graft infection.

## 2. Results

### 2.1. *In Vitro* Studies

*S. aureus* strain SD cells, in the early log phase of growth, were placed in polystyrene plates and grown in static conditions for 24 h. As observed by light microscopy, the growing biofilm covered 12% ± 5% of the surface area after one day and increased to 70% ± 30% after seven days. The mean of viable count recovered from twelve different wells of the same line was within the range of 3 × 10^5^ and 6 × 10^5^ CFU/mL. Tigecycline against the adherent bacteria without FS8 showed MIC and MBC values of 8.00 and 16.00 mg/L. In contrast, tigecycline in presence of FS8 showed MICs (2.00 mg/L) and MBCs (4.00 mg/L) four-fold lower (Table 1).

When MICs and MBCs were determined according to the procedures outlined by the CLSI the experiments showed values four to eight times lower.

### 2.2. *In Vivo* Studies

None of the animals included in the uncontaminated control group had microbiological evidence of graft infection. On the contrary, all 30 rats included in the untreated control group demonstrated evidence of graft infection, with quantitative culture results showing 7.5 × 10^6^ ± 2.1 × 10^6^ CFU/mL.

The rat groups treated with tigecycline showed the lowest bacterial numbers (4.4 × 10^4^ ± 1.2 × 10^4^ CFU/mL). FS8-treated group showed also a good activity and significant differences to the control group with a bacterial numbers of 6.8 × 10^4^ ± 2.0 × 10^4^ CFU/mL. A stronger inhibition of bacterial growth was observed in rats treated with a combined FS8 and tigecycline therapy than in those that were singly treated with bacterial numbers of 10^1^ CFU/mL graft (see Table 2). In particular, the combination proved to be effective in reducing bacterial counts to levels comparable with those observed in uninfected animals.

### 2.3. Peptide Binding to Dacron Graft

The quantity of FS8 bound to Dacron graft was deduced from the unbound peptide concentration in the soaking solution determined by reverse phase HPLC analysis. The results showed that FS8 binds to the grafts with an average amount of 0.67 ± 0.04 μg/cm^2^ (Table 3).

Finally, none of the animals included in any group died or had clinical evidence of drug related adverse effects, such as local signs of perigraft inflammation, anorexia, vomiting, diarrhea, and behavioral alterations.

## 3. Discussion

The vascular graft bacterial infections are associated with high morbidity that may lead to prolonged hospitalization, organ failure, amputation, and death and exert significant financial burden on the healthcare system [29,30].

The ability of *S. aureus* to attach to the foreign body surface and develop a biofilm is an important determinant of resistance to antibiotic prophylaxis. Antimicrobial compounds did not generally demonstrate significant activity in established biofilms due to either a lack of penetration, drug inactivation, or due to the state of bacterial cell division within the biofilm [4–31].

In order to prove efficacy in preventing bacterial adhesion and biofilm formation *in vivo* and *in vitro* a RIP derivative, FS8, was used. For comparison purposes, the antibiotic tigecycline was chosen for its current utilization in clinical practice against staphylococci [17–19,32].

At first we established an *in vitro* system to elucidate the bactericidal activity of tigecycline with or without FS8 in a planktonic model and in an adherent-cell biofilm model. Afterwards, we used a preclinical surgical implant infection in rats that involved the inoculation of MRSA strain in the presence of a collagen-sealed polyester vascular grafts implanted into the subcutaneous pocket surgically placed in the back of these animals.

Our *in vitro* studies showed that biofilm was strongly affected by the presence of FS8, and in its presence, tigecycline showed MICs and MBCs much lower than those obtained in the absence of FS8. These results were confirmed by the *in vivo* experiments, where the use of FS8-graft-bonded with or without combination with the conventional antibiotic caused significantly lower bacterial loads. In fact, similarly to tigecycline, FS8 caused a significant reduction in bacterial load on the vascular graft tissue when compared with control untreated animals and when tigecycline and FS8 were combined resulted in significant bacterial growth inhibition even if high concentrations of organisms are locally inoculated in the area of prosthesis implantation. In summary, not only did FS8 by itself reduce bacterial load, it also enhanced the effect of tigecycline. It could be hypothesized, from our data, that the positive interaction of FS8 with tigecycline could explain by the ability of FS8 and the others RIP analogs to inhibit the production of staphylococci virulence factors. In particular *S. aureus* produces a large number of cell surface-associated components and secreted products that include adhesins, enzymes, toxins, capsular polysaccharides, and other gene products that facilitate tissue colonization, tissue destruction, or immune evasion, which are coordinately regulated by large numbers of regulatory networks [33,34], which controls the expression of genes involved in pathogenesis, metabolic processes, antibiotic resistance, and biofilm formation [35].

We evaluated, in our *in vitro* study, the quantity of FS8 bound to Dacron graft and the results showed that a very small amounts of FS8 bound to the graft, so we may exclude that the activity of FS8 is merely due to its physicochemical property (e.g., amphiphilicity). When tested alone, both tigecycline and FS8 showed to be effective without any toxicity and drug related adverse effects.

## 4. Materials and Methods

### 4.1. Organisms

Methicillin-susceptible (MS) *S. aureus*, strain Smith diffuse, kindly provided by Dr. N. Balaban (Department of Biomedical Sciences, Division of Infectious Diseases, School of Veterinary Medicine, Tufts University, North Grafton, MA 01536, USA) was used. This is a highly encapsulated, slime producing strain with exopolysaccharides, which are antigenically identical to many clinical *S. aureus* strains tested.

### 4.2. Antimicrobial Agents

Preparation of sterile stock solutions of tigecycline (Wyeth Lederle, Aprilia, LT, Italy) was performed according to the manufacturer’s instructions. The antibiotic concentrations used in these experiments corresponded to tigecycline plasma concentrations achievable in humans [36].

RIP and FS8 (Tyr-Ala-Pro-Trp-Thr-Asn-Ala-NH_2_, Figure 1) were synthesized by the solid-phase method on a Rink amide-AM resin (1% DVB, 100–200 mesh, GLS Biochem Ltd., Shanghai, China) using the Fluorenylmethyloxycarbonyl (Fmoc) chemistry.

*N*-α-Fmoc-protected amino acids, 1-hydroxybenzotriazole (HOBt), *O*-Benzotriazole-*N*,*N*,*N*′,*N*′-tetramethyl-uronium-hexafluoro-phosphate (HBTU) were purchased from GLS Biochem Ltd. (Shanghai, China); Kaiser test kit, piperidine, *N*,*N*-diisopropylethylamine (DIPEA), triisopropylsilane (TIS), trifluoroacetic acid (TFA), dimethylformamide (DMF), methanol, and anhydrous diethyl ether, were purchased from Sigma-Aldrich (St. Louis, MO, USA). The following side-chain-protecting groups of the amino acids were used: tBu (Tyr and Thr), Boc (Trp), Trt (Asn).

The peptide chain was elongated in consecutive cycles of deprotection and coupling. Before of each deprotection step the resin was washed with DMF (2×), then the removal of the Fmoc groups was carried out adding 25% piperidine in DMF to the resin for 30 min. After the deprotection, the resin was washed with DCM (3×) and DMF (3×). The peptide elongation steps were performed with 3 equivalents of the protected amino acid, HOBt (3 equiv.), HBTU (3 equiv.), and DIPEA (6 equiv.) for 2 h. After the coupling, the resin was washed with DMF (3×) and DCM (3×) and the completeness of the reaction was confirmed by a negative Kaiser test of small sample of the resin. If the completion tests were positive, the coupling was repeated. After the final deprotection, the resin was washed with DMF (3×), DCM (3×), and MeOH (3×), dried under vacuum and then stirred with a mixture of TFA:TIS:H_2_O (95:2.5:2.5) for 3 h. After this time, the resulting solution was filtered to remove resin beads and the peptide was precipitated with cold Et_2_O. The crude product was further purified using reversed phase HPLC on a Waters 600 HPLC (Waters Co., Milford, MA, USA) equipped with an X-Bridge^®^ Prep BEH130 C18, 5 μm (10 × 250 mm) column, with Waters 2996 PDA detector, using a solvent system of H_2_O/CH_3_CN (0.1% TFA) in the form of a linear gradient of CH_3_CN, from 5% to 30% in 10 min, from 30% to 35% in 15 min, from 35% to 90% in 5 min, and from 90% to 5% in 10 min, and a flow rate of 3 mL/min, yielding the peptide as trifluoacetate salts. The purity of the peptide was further analyzed by reverse phase HPLC using an X-Bridge BEH130 C18 (Waters Co., Milford, MA, USA), 5 μm, 4.6 × 250 mm column and a Waters 2996 PDA detector (280 nm and 220 nm) [30]. The identity of the peptide was confirmed by a MS spectra recorder using a LC–MS/MS system consisted of an LCQ (Thermo Finnigan, San Jose, CA, USA) ion trap mass spectrometer equipped with an electro-spray ionization (ESI) source (Table 3). The capillary temperature was set at 300 °C and the spray voltage at 4.25 kV. The fluid was nebulized using nitrogen (N_2_) as both the sheath gas and the auxiliary gas.

### 4.3. Adherent Biofilm Formation for Susceptibility Testing

To develop biofilms, 50 μL of Triptic Soy broth (TSB) (Oxoid S.p.A., Milan, Italy) containing 10^6^ Colony Forming Unit (CFU)/mL of bacteria were added under aseptic conditions to each well of a tissue culture-treated polystyrene 96-well plate (Becton-Dickinson, Milano, Italy) containing 150 μL of TSB-2% glucose. After 24 h incubation at 37 °C, the growth medium was discarded and each well was washed three times with Phosphate-Buffered Saline (PBS) under aseptic conditions to eliminate unbound bacteria. To evaluate the formation of adherent biofilm, the remaining attached bacteria were fixed with 0.2 mL of 99% methanol per well, and after 15 min plates were emptied and left to dry. Following, plates were stained for 5 min with 0.2 mL of 2% crystal violet used for Gram staining per well. Excess stain was rinsed off by placing the plate under running tap water [37,38]. The plates were air-dried and the dye bound to the adherent cells was resolubilized with 0.2 mL of 33% volume-to-volume (*v*/*v*) glacial acetic acid per well. The optical density (OD) of each well was determined photometrically at 570 nm by using the MR 700 Microplate Reader (Dynatech Laboratories, Guernsey, UK). The 0.00 value (negative control) was determined for every plate measuring the optical density of a well filled with PBS solution. The cut-off OD for the microtiter-plate test was defined as three standard deviations above the mean OD of the negative control. The same experiment was performed two times: (i) with and (ii) without addition of 10 μg of FS8 in a total volume of 10 μL Mueller-Hinton (MH) broth in each well. Tests were performed in triplicate. Biofilms were also observed by lights microscopy. Tests were performed in duplicate.

### 4.4. Susceptibility Testing with Adherent Cells

For use in the biofilm test, the MIC and MBC, respectively were determined with modifications. Biofilms were washed with phosphate-buffered saline (PBS) in order to discard unbound bacteria. Subsequently, serial two-fold dilution of antibiotic, in MH broth, was added to wells containing adherent organisms. The polystyrene plates were incubated for 18 h at 37 °C in air. The MIC was taken as the lowest tigecycline concentration at which observable growth was inhibited. To determine the MBC, the MH broth containing FS8 was removed from each well and replaced with antibiotic-free MH broth; the plates were incubated again for 18 h at 37 °C in air. The MBC was taken as the lowest concentration of tigecycline that resulted in no bacterial growth following removal of the drug [10]. In addition, to investigate the effect of FS8 pre-treatment on bacterial antibiotic susceptibility, MIC and MBC were determined after pre-treatment of cells for 30 min with 10 μg FS8 in 10 μL MH broth/well.

### 4.5. Susceptibility Testing with Planktonic Bacteria

MICs and MBCs were determined according to the procedures outlined by the Clinical and Laboratory Standards Institute (CLSI) [39]. Experiments were performed in triplicate.

### 4.6. Peptide Binding to Dacron Graft

To determine the peptide concentration impregnated on the graft, 1 cm^2^ slices of collagen-sealed Dacron were soaked at room temperature in peptide solution in triplicate for 30 min (1 mL of saline per graft, containing 10 mg/L of the peptide). Following the incubation period, the Dacron was removed, and the-peptide concentration in solution was determined using HPLC reverse phase (see above). The unbound peptide concentration was calculated by area integration of the UV-absorbing peak (220 nm and 280 nm) and compared with standard curves of the reference peptide [28].

Adult male Wistar rats (weight range, 250 to 320 g) were used for all the experiments. The study was approved by the Animal Research Ethics Committee of the I.N.R.C.A. I.R.R.C.S., Italy.

The study included a control group, a contaminated group that did not receive any antibiotic prophylaxis and three contaminated groups that received (i) intraperitoneal tigecycline (2 mg/Kg), (ii) FS8-soaked graft, and (iii) daptomycin plus FS8-soaked graft, respectively. Each group included 15 animals. The infection burden was evaluated by using sonication and quantitative agar culture. Each group included 15 animals and, to verify the results, the experiments were performed in duplicate. In the statistical analysis, the data were merged and referred to all 30 animals from each pair of groups. Rats were anesthetized with ether, the hair on the back was shaved and the skin cleansed with 10% povidone-iodine solution. Immediately before surgical procedure each group received a single dose of the above-mentioned intraperitoneal compounds at the established dosages. One subcutaneous pocket was made on each side of the median line by a 1.5 cm incision. Aseptically, 1 cm^2^ sterile collagen-sealed polyester vascular grafts were implanted into the pockets. In the groups locally treated with the antibiotic-soaked graft, binding was obtained immediately before implantation by soaking grafts for 30 min in a sterile solution of the 10 mg/L FS8. The pockets were closed by means of skin clips and a saline solution (1 mL) containing 2 × 10^7^ CFU/mL staphylococcal strain was inoculated on to the graft surface using a tuberculin syringe to create a subcutaneous fluid-filled pocket. Immediately before the surgical procedure, two groups received a single dose of intraperitoneal tigecycline to the established dosage. The animals were returned to individual cages and thoroughly examined daily. Based on experiments demonstrating peak bacterial growth and biofilm formation within 72 h (data not shown), all grafts were explanted at seven days following implantation. Toxicity was evaluated on the basis of the presence of any drug related adverse effects, *i.e.*, local signs of perigraft inflammation, anorexia, weight loss, vomiting, diarrhea, fever, and behavioral alterations.

### 4.7. Assessment of the Infection

The explanted grafts were placed in sterile tubes, washed in sterile saline solution, placed in tubes containing 10 mL of PBS solution and sonicated for 2 min to remove the adherent bacteria from the grafts. Quantization of viable bacteria was performed by culturing serial 10-fold dilutions (0.1 mL) of the bacterial suspension on blood agar plates. All plates were incubated at 37 °C for 48 h and evaluated for the presence of the staphylococcal strain. The organisms were quantified by counting the number of CFU per plate. The limit of detection for this method was approximately 10 CFU/mL.

### 4.8. Statistical Analysis

MIC values are presented as the geometric mean of three separate experiments. Quantitative culture results regarding the *in vivo* experiments were presented as mean ± S.D. of the mean and the statistical comparisons between groups were made using analysis of variance (ANOVA) on the log-transformed data with Tukey-Kramer Honestly Significant Difference Test. Significance was accepted when the *p* value was ≤0.05.

## 5. Conclusions

In conclusion our preliminary study suggests FS8, for its ability to affect biofilm formation as well its property to be an antibiotic enhancer, as alternative or additional agent to use in conjunction with conventional antimicrobial for prevention of staphylococcal biofilm related infection.

We are aware of problems, in spite of several positive facts associate with antimicrobial peptides, related to few data available on the unknown *in vitro* and *in vivo* toxicities and cost of their production on a large scale.

For solving the biofilms associated infections there are necessary different therapeutical strategies and further medical research on biofilms with the purpose to discover efficient methods/combinations, ecologic alternatives to antibiotics; we believe that the quorum sensing inhibitors FS8 can be such an alternative.

## Figures and Tables

**Table 1:** *In vitro* antimicrobial activity of tigecycline against *S. aureus* strain Smith adherent cells with or without FS8 pre-treatment.

	Biofilm	
	
Agent	MIC (μg/mL)	MBC (μg/mL)
Tigecycline	8.00	16.00
Tigecycline *	2.00	4.00

* FS8 pre-treatment (10 μg FS8 in 10 μL MH broth/well).

**Table 2:** Activity of tigecycline and FS8 against *Staphylococcus aureus*, strain Smith diffuse (SD) in a subcutaneous rat pouch model of infection.

Group a	Graft-bonded drug b	Intraperitoneal preoperative drug c	Quantitative graft culture (CFU/mL) d	*p*-value (comparison with untreated control group)	*p*-value (comparison with double-treated group)
Uncontaminated control		-	<10		
Untreated control		-	7.5 × 10^6^ ± 2.1 × 10^6^		
Group 1	-	Tigecycline	4.4 × 10^4^ ± 1.2 × 10^4^	<0.001	<0.001
Group 2	FS8	-	6.8 × 10^4^ ± 2.0 × 10^4^	<0.001	<0.001
Group 3	FS8	Tigecycline	3.7 × 10^1^ ± 0.7 × 10^1^	<0.001	<0.001

a *Staphylococcus aureus*, strain Smith diffuse (SD);

b The Dacron graft segments were impregnated with 10 mg/L of FS8;

c Each rat received intraperitoneally 2 mg/Kg of tigecycline;

d The limit of detection for the method was ≤10 CFU/mL.

**Table 3:** Analytical data of FS8.

RP-HPLC retention time (min)	Purity (%)	Mass		Binding to the grafts (μg/cm^2^)
		Calculated	Found	
14.43	97.85	836.393	836.388	0.67 ± 0.04
